# Target product profiles for the diagnosis of *Taenia solium* taeniasis, neurocysticercosis and porcine cysticercosis

**DOI:** 10.1371/journal.pntd.0005875

**Published:** 2017-09-11

**Authors:** Meritxell Donadeu, Anna S. Fahrion, Piero L. Olliaro, Bernadette Abela-Ridder

**Affiliations:** 1 The University of Melbourne, Faculty of Veterinary and Agricultural Sciences, Veterinary Clinical Centre, Werribee, Victoria, Australia; 2 Initiative for Neglected Animal Diseases (INAND), Midrand, South Africa; 3 Department of Control of Neglected Tropical Diseases, World Health Organization, Genève, Switzerland; 4 Special Programme for Research and Training in Tropical Diseases (TDR), World Health Organization, Genève, Switzerland; Universidad Peruana Cayetano Heredia, PERU

## Abstract

Target Product Profiles (TPPs) are process tools providing product requirements to guide researchers, developers and manufacturers in their efforts to develop effective and useful products such as biologicals, drugs or diagnostics. During a WHO Stakeholders Meeting on *Taenia solium* diagnostics, several TPPs were initiated to address diagnostic needs for different stages in the parasite’s transmission (taeniasis, human and porcine cysticercosis). Following the meeting, draft TPPs were completed and distributed for consultation to 100 people/organizations, including experts in parasitology, human and pig cysticercosis, diagnostic researchers and manufacturers, international organizations working with neglected or zoonotic diseases, Ministries of Health and Ministries of Livestock in some of the endemic countries, WHO regional offices and other interested parties. There were 53 respondents. All comments and feedback received were considered and discussions were held with different experts according to their area of expertise. The comments were consolidated and final TPPs are presented here. They are considered to be live documents which are likely to undergo review and updating in the future when new knowledge and technologies become available.

## Author summary

Diagnosing the different manifestations of *Taenia solium* (taeniasis and neurocysticercosis in humans, and pig cysticercosis) presents many challenges. The current paper describes the Optimal and Minimal attributes of the diagnostic tests that would be fit for a variety of diagnostic purposes, presented in the form of Target Product Profiles (TPPs). Four TPPs were drafted, distributed for consultation, and the feedback received was consolidated to produce the final versions presented here.

## Introduction

Neurocysticercosis (NCC) is a Neglected Tropical Disease caused by infection with the cestode parasite *Taenia solium*. NCC is endemic in many low- and middle-income countries [1] and is the most frequent cause of acquired epilepsy in those countries and probably the world [2]. In endemic areas, 29% of neurological seizures are attributable to NCC [3].

The lifecycle of *T*. *solium* involves two hosts. Humans harbour the sexually reproducing mature tapeworm in the small intestine (taeniasis). Eggs released with the faeces are infectious if eaten by pigs in which the larval parasite (cysticercus) encysts in the muscle tissues and brain, causing porcine cysticercosis. Ingestion of poorly cooked, infected pig meat by humans leads to growth of the tapeworm, completing the lifecycle. Eggs in the faeces of a patient infected with the adult tapeworm are also infectious if they are ingested by humans, in which case the cysticerci may lodge in muscle and other tissues, including the central nervous system causing NCC.

Symptoms of NCC vary depending, among other things, on the number, size and location of the cysts. They include headache, blindness, convulsions, epilepsy and death. The disease usually affects very poor communities, where pigs live in close contact with humans, and hygiene, sanitation and education are limited. The disease has a very strong social impact; epilepsy sufferers are often stigmatised, especially if sufferers are women and girls, as it is linked with witchcraft.

Diagnosis of NCC is complicated, and neuroimaging is frequently required for a definite diagnosis [4, 5]. Currently there are no standard treatment guidelines for NCC, and treatment is tailored to the individual cases [2, 6, 7], depending on factors such as location and viability of the cysts. Therapeutic approaches might include symptomatic therapy, anthelmintic treatment, or surgery, and often more than one of these options is needed [8]. The administration of anthelmintic drugs may elicit or increase pre-existing cerebral oedema and therefore is contraindicated in cases with cysticercotic encephalitis, increased intracranial pressure and subarachnoid NCC in close proximity to blood vessels [6, 8], thus it is very important to have the neuroimaging diagnostic before starting the treatment of living cysts. Suitable imaging diagnostic facilities are not readily available in the poor rural communities where the disease is endemic, and there is a need for better diagnosis tools to identify patients in rural and remote communities with viable cysts that need to be referred to neuroimaging before treatment.

There are several options for NCC disease control at population level, aimed at breaking the parasite’s transmission cycle. They include identification and treatment, or mass treatment for taeniasis (infection in humans with the adult tapeworm), chemotherapeutic treatment of porcine cysticercosis, pig vaccination, improvements in pig housing and management, meat inspection, improved human hygiene and sanitation, health education, etc. [9]. Efforts to control the disease have usually included several of these options, with the particular choices varying depending on the setting and the resources available.

One of the challenges faced by efforts to control *T*. *solium* is a need for better diagnostic tools to monitor the outcome of control efforts [10]. Evaluation of control programs can be achieved by monitoring taeniasis or monitoring porcine cysticercosis. Monitoring control programs through changes in the prevalence of NCC is not recommended due to the persistence of NCC infections over long periods [10]. For *Taenia solium*, and generally for programs addressing neglected tropical diseases targeted for control and elimination [11], it is crucial to have high-quality, low-cost diagnostic tools available and deployable in low-resource settings.

In December 2015 the World Health Organization (WHO) Department of Control of Neglected Tropical Diseases (WHO/NTD) and the Special Programme for Research and Training in Tropical Diseases (WHO/TDR) convened a stakeholder meeting [12] to discuss the difficulties with the diagnosis of *T*. *solium* infections, to coordinate and harmonize the search for appropriate diagnostic tests and to overcome the challenges in doing so. During the meeting, diagnostic priorities for different stages of the parasite’s transmission (taeniasis, human and porcine cysticercosis) were defined, and it was decided to initiate several Target Product Profiles (TPPs). An important step early in the process of designing diagnostic tools is defining their essential features, to allow researchers and diagnostic manufacturers to develop solutions that meet the needs in specific settings and for defined purposes. A TPP can be described as an important strategic document doing exactly this—describing key characteristics of a product. TPPs are used by the pharmaceutical and diagnostic industries, but also by organizations working on developing tools for the control of neglected diseases. Here we describe the development of four TTPs for *T*. *solium* taeniasis/cysticercosis.

## Methodology

Following the Stakeholder Meeting on *T. solium* Taeniasis/Cysticercosis Diagnostic Tools held at WHO headquarters, Geneva in December 2015, four TPPs were prioritised and progressed, and final drafts were elaborated. The attributes selected were based on previously published diagnostic TPPs produced by organizations such as FIND (www.finddx.org) and PATH (www.path.org), as well as the attributes suggested by the WHO [13]. Two TPPs were produced for taeniasis, one for NCC and one for porcine cysticercosis, as follows.

1. Taeniasis in humans caused by *T*. *solium*: A Point-of-care test that could be used for surveillance, could be used at the initial stages of a control program, as well as for Diagnosis & Treatment.
2. Taeniasis in humans caused by *T*. *solium*: A specific test (various platforms). Similar to number 1, but a test that could be used at later stages of a control program, when specificity and sensitivity are critical. This test could also be used for Diagnosis & Treatment.
3. Human NCC: A Point-of-care test, to identify symptomatic patients with viable cysts that need to be referred for confirmation by brain imaging.
4. Porcine cysticercosis (various platforms) test to be used to monitor control programs.

TPPs for other use cases, such as screening of human populations, or detection of positive pigs through the pork chain, were not prioritised at this point in time.

The draft TPPs were circulated to an extensive group of stakeholders for consultation. These included a wide range of experts in parasitology, human and pig cysticercosis, diagnostic experts, diagnostic manufacturers, organizations working with neglected diseases, organizations working with zoonotic diseases, some Ministries of Health and Directors of Veterinary services, WHO regional offices, as well as other interested parties. Efforts were made to ensure individuals and/or organizations from all *T*. *solium* endemic regions [1] were included. In total, 100 persons/institutions were contacted directly, and those receiving the drafts were requested to pass them to their colleagues if they thought appropriate. The experts/organizations contacted included 22 from Africa, 21 from the Americas, 28 from Asia-Pacific, 21 from Europe and 8 from International Organizations. Experts were given one month to provide feedback. The consultation took place during March 2017, and it was extended until Mid-April 2017.

All comments and suggestions received were considered. Clarifications and follow up discussions were held with almost all contributors according to their area of expertise, to reach a consensus or arrive at a decision in relation to the various aspects of the final TPPs. In many cases, compromises needed to be made in order to keep the TPP attributes realistic and to account for the different opinions and needs as best as possible.

## Results and discussion

**Fig 1:**
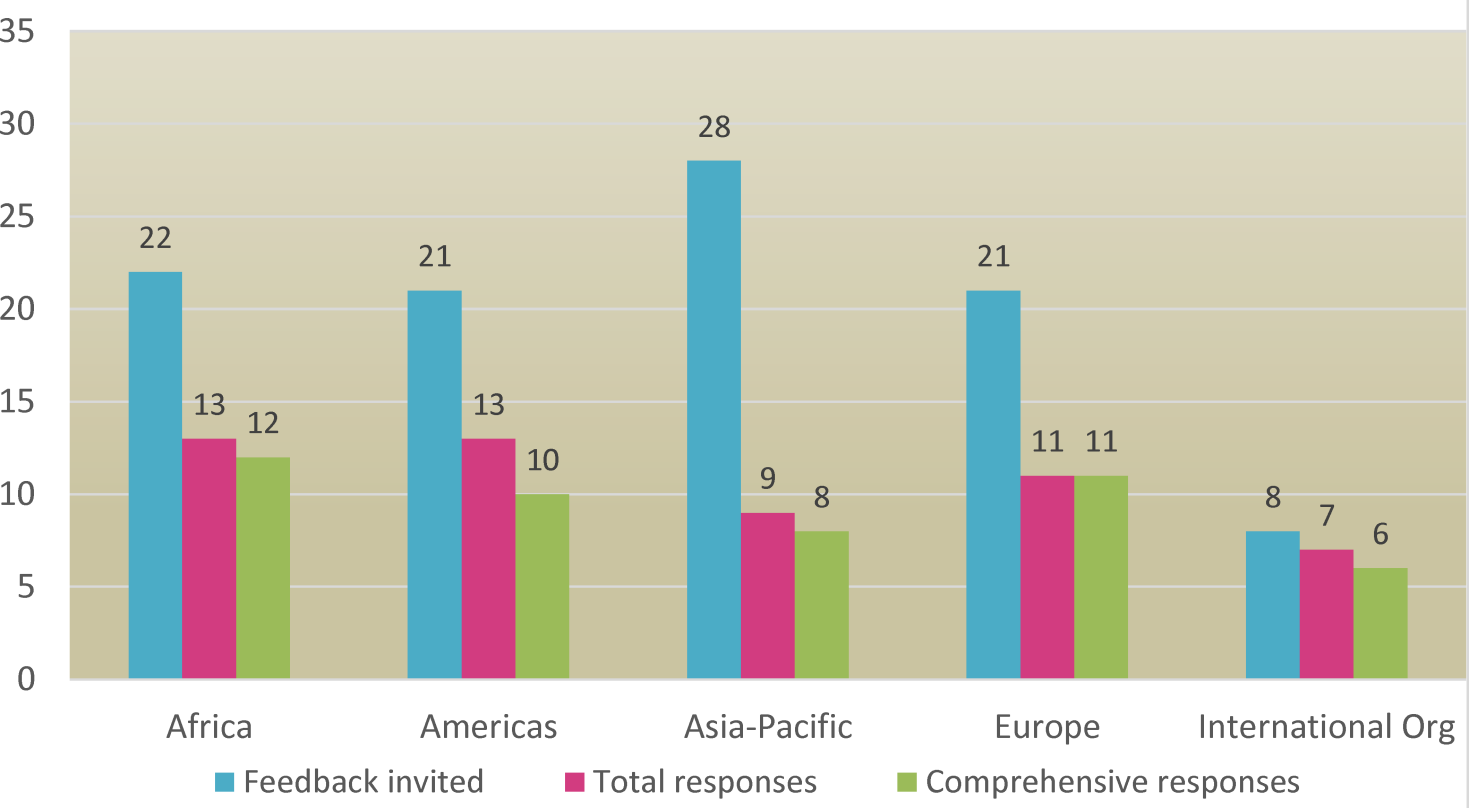
Number of people invited to provide feedback and number of responses received. Of the 53 total responses received, 47 were comprehensive, and 6 were void.

A total of 53 replies were received: 13 from Africa, 13 from the Americas, 9 from Asia-Pacific, 11 from Europe, and 7 from International Organizations (Fig 1). Six of them were considered void, as they did not include comments, but the respondents said they were not able to contribute due to various factors such as change of research area, lack of expertise, conflict of interest, or other circumstances. On the remaining 47 comprehensive contributions, of which 12 (26%) were from Africa, 10 (21%) from the Americas, 8 (17%) from Asia-Pacific, 11 (23%) from Europe and 6 (13%) from International Organizations. Feedback was received not only from disease experts, but also from diagnostic manufacturers (1), from a veterinary product distribution company in Africa (1), and international organizations. In total there were 4 industry responses (1 Africa, 1 America, 2 Europe), 3 government and officials responses (2 Africa, 1 Americas), 1 response from a local NGO (Asia-Pacific), 6 responses from International Organizations (including 3 international NGOs), and 33 from research organizations and academia. Many of the research organizations were also government facilities and some of the respondents were government officials. Similarly, many of the professionals working in research organizations or academia also work as health professionals, for example some of the neurologists in research organizations also work as health practitioners.

The final TPPs are presented in Tables 1–4. While the TPPs are considered to be final at this stage, they are regarded as being live documents that will require review, possibly after 4–5 years as knowledge develops and technologies advance.

**Table 1:** TPP for a point-of-care test for the detection of *Taenia solium* taeniasis in humans.

	Characteristic or attribute	Optimal	Minimal
1	Context and product overview		
1.1	Indication	For the detection of current infection in humans with *Taenia solium* non-gravid and mature (gravid) tapeworm. To test negative within 6 days following successful treatment for taeniasis.	Detection of infection with mature *Taenia solium* tapeworm in humans. To test negative within 6 days following successful treatment for taeniasis.
1.2	Intended use (use case; in order of priority)	1- Monitoring of *T*. *solium* control interventions (all stages of a control program). 2- Surveillance of human taeniasis due to *T*. *solium*.	1- Surveillance of human taeniasis. 2- Monitoring taeniasis at the initial stages of a control program.
1.3	Target populations	Humans > 2 years of age	Humans > 2 years of age
1.4	Target setting for deployment	Cysticercosis endemic or suspected endemic countries	Cysticercosis endemic or suspected endemic countries
1.5	Location of use (infrastructure level)	Basic infrastructure including community health centers, households and outdoor conditions (Tier 2)^a^.	Basic infrastructure including community health centers (Tier 2)^a^.
1.6	Target user	Community health workers, trained lay persons and trained community volunteers.	Community health workers
2	Design and operational characteristics		
2.1	Format (product presentation)	Rapid Diagnostic Test, multiple formats accepted. All reagents needed included as a kit.	Rapid Diagnostic Test (RDT), multiple formats accepted. All reagents needed included as a kit.
2.2	Portability	Portable	Portable
2.3	Target analyte (diagnostic marker)	To be determined. Specific antigen/epitope for the presence of *T*. *solium*.	To be determined. Specific antigen/epitope for the presence of *Taenia* spp.
2.4	Sample type and quantity	1- Stool (0.05–0.3 g)—fresh, frozen or fixed OR 2- Potentially rectal swab	1- Stool (0.05–2 g)—fresh or frozen
2.5	Sample handling and preparation	≤ 2 steps	≤ 2 steps
2.6	Steps to test result	≤ 3 steps	≤ 4 steps
2.7	Nature of result	Qualitative	Qualitative
2.8	Time to results	15 minutes or less	30 minutes or less
2.9	Results records	Test methodology enables clear association between sample ID and result	Test methodology enables clear association between sample ID and result
2.10	Ease of results interpretation	Visual readouts without data interpretation	Visual readouts without data interpretation
2.11	Duration of valid result	≥ 30 min	≥ 10 min
2.12	Throughput	> 100 samples per day per operator	> 30 samples per day per operator
2.13	Equipment/Instrumentation format and complexity	Rapid Diagnostic Test with minimal user steps	Rapid Diagnostic Test with minimal user steps. Timing device required.
2.14	Ease of use	One or no timed steps; instructions should be intuitive, include diagram of method and results interpretation, and minimal words, maximum 1 page (per language). Should include instructions for safe sample manipulation and disposal. No need of transfer small volumes of reagent/sample, no need to measure precise volume of reagent or weight samples. Instructions for use in English, French, Spanish, Portuguese.	Two or fewer timed steps; instructions should include diagram of method and results interpretation. Only few words, maximum 2 pages. Should include instructions for safe sample manipulation and disposal.
2.15	Test-specific training requirements	Minimal: 1 day for lay person, 1/2 day for health care worker familiar with the type of test.	Minimal: 1 day
2.16	Supplies needed	None	Minimal supplies to prepare the sample, packaged as a kit.
2.17	Refrigeration required (storage and transportation)	None required	Store between 2–30°C
2.18	Power requirements	Not required	None or battery powered
2.19	Water requirements	Self-contained kit. Not required.	Self-contained kit. Not required.
2.20	Operating temperature	10–40°C	20–35°C
2.21	Waste management (hazardous materials/chemicals)	Does not include material that cannot be disposed of safely in the normal health community centers. Minimal or no hazardous materials, per WHO and country standards.	Does not include material that cannot be disposed of safely in the normal health community centers. Minimal or no hazardous materials, per WHO and country standards.
2.22	Quality Control	Internal control. Positive and negative external controls supplied with the kit; to be used if deemed necessary. One set of external controls provided per box of tests.	Internal control
2.23	Calibration	Not required	Not required
3	Performance		
3.1	Species differentiations	*T*. *solium* only (species specific)	Target is *T*. *solium*, but could cross react with other *Taenia* spp
3.2	Analytic sensitivity / limit of detection	Positive test would detect the presence of 1 tapeworm (mature or non-gravid).	Positive test would detect the presence of 1 tapeworm
3.3	Clinical sensitivity	≥ 95%	≥ 99%
3.4	Clinical specificity	≥ 99%	≥ 80%
3.5	Reproducibility & robustness	Replicate of weak positive, classify the same > 95% of the time.	Replicate of weak positive, classify the same > 90% of the time.
3.6	Comparative reference method	Nested PCR developed by Mayta et al, 2008 [14].	Nested PCR developed by Mayta et al, 2008 [14].
3.7	Shelf life, stability	36 months at temperatures between 2–30°C; stable for 2 weeks at 50°C.	24 months at temperatures between 2–30°C; stable for 2 weeks at 40°C.
4	Patient access / commercialization		
4.1	Relevant range of cost (price to end-user)	≤ 0.5 USD	≤ 2 USD per test. Below cost of treatment.
4.2	Test pack size	100 tests/pack	100 tests/pack
4.3	Supply: channels to market	To be determined	To be determined
4.4	Supply: service and support	To be determined	To be determined
4.5	Product registration path	As per local regulations. ISO 13485 for the manufacturing company.	As per local regulations. ISO 13485 for the manufacturing company.

^a^ Tier definitions used as described in Pai et al, 2012 [15]

**Table 2:** TPP for a specific test for the detection of *T*. *solium* taeniasis in humans (various platforms).

	Characteristic or attribute	Optimal	Minimal
1	Context and product overview		
1.1	Indication	For the detection of current infection in humans with *Taenia solium* non-gravid and mature (gravid) tapeworm. To test negative within 6 days following successful treatment for taeniasis.	Detection of infection with mature *Taenia solium* tapeworm in humans. To test negative within 6 days following successful treatment for taeniasis.
1.2	Intended use (use case; in order of priority)	1- Monitoring of *T*. *solium* control interventions (all stages of a control program). 2- Surveillance of human taeniasis due to *T*. *solium*.	1- Monitoring of *T*. *solium* control interventions (all stages of a control program). 2- Surveillance of human taeniasis due to *T*. *solium*.
1.3	Target populations	Humans > 2 years of age	Humans > 2 years of age
1.4	Target setting for deployment	Cysticercosis endemic or suspected endemic countries	Cysticercosis endemic or suspected endemic countries
1.5	Location of use (infrastructure level)	Health centres with basic laboratory facilities (Tier 2)^a^.	Diagnostic facilities (independent, or included in a District hospital for example) (Tier 3 or 4)^a^. Research laboratories.
1.6	Target user	Health care workers with laboratory training	Skilled laboratory technicians
2	Design and operational characteristics		
2.1	Format (product presentation)	Technologies providing high specificity and ease of use: Patterned paper, Lab-on-a-Chip (LOC), Lab-on-Compact Disc (LOCD), field deployable nucleic acid amplification test (NAAT), etc.	Any format meeting the performance requirements
2.2	Portability	Portable	Non-portable
2.3	Target analyte (diagnostic marker)	To be determined. Specific antigen/epitope or nucleic acid for the presence of *T*. *solium*.	To be determined. Specific antigen/epitope for the presence of *T*. *solium*.
2.4	Sample type and quantity	1- Stool (0.05–0.3 g)—fresh, frozen or fixed OR 2- Potentially rectal swab	1- Stool (0.05–2 g)—fresh or frozen
2.5	Sample handling and preparation	≤2 steps. Samples stable when refrigerated (2–8°C) for 2 days. Samples can be fixed and used for up to 24 months.	≤ 3 steps. Samples stable when refrigerated (2–8°C) for 2 days.
2.6	Steps to test result	≤ 3 steps	Many steps
2.7	Nature of result	Qualitative	Qualitative
2.8	Time to results	Same day result, < 60 mins.	< 24 hours
2.9	Results records	Test methodology enables clear association between sample ID and result	Test methodology enables clear association between sample ID and result
2.10	Ease of results interpretation	Easy interpreted by minimally skilled health workers. No possibility for subjective interpretation.	Some basic calculations might be required
2.11	Duration of valid result	≥ 30 min	≥ 10 min
2.12	Throughput	> 300 samples per day	> 180 samples per day
2.13	Equipment/Instrumentation format and complexity	Field-deployable, low complexity equipment.	Basic laboratory equipment such as ELISA readers, water bath, vortex mixers and others might be required.
2.14	Ease of use	Two or fewer timed steps; instructions should be intuitive, and include diagram of method and results interpretation. Should include instructions for safe sample manipulation and disposal. Max 2 pages. Instructions for use in English, French, Spanish, Portuguese.	Five or fewer timed steps; instructions should include diagram of method and results interpretation. Should include instructions for safe sample manipulation and disposal. Max 4 pages.
2.15	Test-specific training requirements	Less than 3 days	Several days
2.16	Supplies needed	Minimal supplies to prepare the sample, packaged as a kit.	Distilled water, pipettes and tips, timer, laboratory material.
2.17	Refrigeration required (storage and transportation)	None required	Reagents to be kept refrigerated (2–8°C)
2.18	Power requirements	Batteries or no power requirements	Some equipment (if needed) such as ELISA readers and others might require mains power, as per manufacturer recommendations (ensure correct voltage and amperage).
2.19	Water requirements	Self-contained kit. Not required.	Distilled or double de-ionized water
2.20	Operating temperature	10–40°C	20–25°C
2.21	Waste management (hazardous materials/chemicals)	Does not include material that cannot be disposed of safely in the normal health community centers. Minimal or no hazardous materials, per WHO and country standards.	Some moderate hazards permitted (e.g. stopping solutions might contain hazardous substances). Safe disposal of stool samples.
2.22	Quality Control	Industry standards for positive and negative controls	Industry standards for positive and negative controls
2.23	Calibration	Minimal. Not required in the field.	Readers, pipettes and others to be calibrated as per manufacturers recommendations
3	Performance		
3.1	Species differentiations	*T*. *solium* only	*T*. *solium* only
3.2	Analytic sensitivity / limit of detection	Positive test would detect the presence of 1 tapeworm (mature or non-gravid).	Positive test would detect the presence of 1 tapeworm.
3.3	Clinical sensitivity	≥ 95%	≥ 95%
3.4	Clinical specificity	≥ 99%	≥ 99%
3.5	Reproducibility & robustness	Replicate of weak positive, classify the same > 95% of the time	Replicate of weak positive, classify the same > 90% of the time
3.6	Comparative reference method	Nested PCR developed by Mayta et al, 2008 [14].	Nested PCR developed by Mayta et al, 2008 [14].
3.7	Shelf life, stability	36 months at temperatures between 2–40°C	24 months at temperatures between 2–30°C; stable for 2 weeks at 40°C (except for reagents that must be refrigerated: see 2.17).
4	Patient access / commercialization		
4.1	Relevant range of cost (price to end-user)	≤ 0.5 USD per test	≤ 2 USD per test
4.2	Test pack size	100 tests/pack	90 tests/pack
4.3	Supply: channels to market	To be determined	To use existing ELISA suppliers
4.4	Supply: service and support	To be determined	To be determined
4.5	Product registration path a	As per local regulations. ISO 13485 for the manufacturing company.	As per local regulations. ISO 13485 for the manufacturing company.

^a^ Tier definitions used as described in Pai et al, 2012 [15]

**Table 3:** TPP for a point-of-care test for the diagnosis of human neurocysticercosis.

	Characteristic or attribute	Optimal	Minimal
1	Context and product overview		
1.1	Indication	1) To identify patients with symptomatic neurocysticercosis that require referral for imaging and treatment. 2) Post-treatment follow up of cysticercosis patients (specific scenarios).	To identify patients with symptomatic neurocysticercosis that require referral for imaging and treatment.
1.2	Intended use (use case; in order of priority)	1) Supportive diagnosis of symptomatic neurocysticercosis; should not detect transient positives. 2) Post-treatment follow up.	Supportive diagnosis of symptomatic neurocysticercosis. Re-testing at a specific interval might be necessary to differentiate transient positives (see 3.4).
1.3	Target populations	Humans with clinical signs suggestive of NCC	Humans with clinical signs suggestive of NCC
1.4	Target setting for deployment	Cysticercosis endemic or suspected endemic countries	Cysticercosis endemic or suspected endemic countries
1.5	Location of use (infrastructure level)	Basic infrastructure including community health centers, households and outdoor conditions (Tier 2)^a^.	Basic infrastructure including community health centers (Tier 2)^a^.
1.6	Target user	Community health workers, trained lay persons and trained community volunteers; supported by physicians.	Community health workers supported by physicians who provide patient treatment/support.
2	Design and operational characteristics		
2.1	Format (product presentation)	Rapid Diagnostic Test, multiple formats accepted. All reagents needed included as a kit.	Rapid Diagnostic Test, multiple formats accepted such as Lateral flow assay (with cassette).
2.2	Portability	Portable	Portable
2.3	Target analyte (diagnostic marker)	Specific antigen for the presence of active (live) *T*. *solium*.	Specific antigen for the presence of active (live) *T*. *solium*.
2.4	Sample type and quantity	Capillary whole blood (finger stick), serum or saliva—50μl.	Capillary whole blood (finger stick) or serum—50μl.
2.5	Sample handling and preparation	≤ 2 steps	≤ 2 steps
2.6	Steps to test result	≤ 3 steps	≤ 3 steps
2.7	Nature of result	Semi-quantitative or quantitative	Qualitative
2.8	Time to results	15 minutes or less	30 minutes or less
2.9	Results records	Test methodology enables clear association between sample ID and result	Test methodology enables clear association between sample ID and result
2.10	Ease of results interpretation	Easy interpreted by minimally skilled health workers. No possibility for subjective interpretation.	Visual readouts without data interpretation
2.11	Duration of valid result	≥ 30 min	≥ 10 min
2.12	Throughput	50 samples per day	25 samples per day
2.13	Equipment/Instrumentation format and complexity	Rapid Diagnostic Test with minimal user steps	Simple test with minimal user steps. Timing device required.
2.14	Ease of use	One or no timed steps; instructions should be intuitive, include diagram of method and results interpretation, and minimal words, maximum 1 page (per language). Should include instructions for safe sample manipulation and disposal. No need of transfer small volumes of reagent/sample, no need to measure precise volume of reagent or weight samples. Instructions for use in English, French, Spanish, Portuguese.	Two or fewer timed steps; instructions should include diagram of method and results interpretation. Only few words, maximum 2 pages. Should include instructions for safe sample manipulation and disposal.
2.15	Test-specific training requirements	Minimal: 1 day for lay person, 1/2 day for health care worker familiar with the type of test.	Minimal: 1 day
2.16	Supplies needed	None	Minimal supplies to prepare the sample, packaged as a kit.
2.17	Refrigeration required (storage and transportation)	None required	Store between 2–30°C
2.18	Power requirements	Not required	Not required
2.19	Water requirements	Self-contained kit. Not required.	Self-contained kit. Not required.
2.20	Operating temperature	10–40°C	20–35°C
2.21	Waste management (hazardous materials/chemicals)	Does not include material that cannot be disposed of safely in the normal health community centres. Minimal or no hazardous materials, per WHO and country standards.	Does not include material that cannot be disposed of safely in the normal health community centres. Minimal or no hazardous materials, per WHO and country standards.
2.22	Quality Control	Internal control	Internal control
2.23	Calibration	Not required	Not required
3	Performance		
3.1	Species differentiations	*T*. *solium* only	*T*. *solium* only
3.2	Analytic sensitivity / limit of detection	Should detect patients with a single intracranial cysticercus, including both intraparenchymal and extra-parenchymal cysts	Should detect patients with 5 or more parenchymal cysticerci, and detect a single ventricular or subarachnoid cysticercus.
3.3	Clinical sensitivity	≥ 99%	≥ 98%
3.4	Clinical specificity	≥ 95%	≥ 90%. Test might have to be repeated three months apart, to confirm it is not a transient reaction. However, depending on the history and severity of the signs, the physician might decide to refer the patient without repeat testing.
3.5	Reproducibility & robustness	Replicate of weak positive, classify the same > 95% of the time.	Replicate of weak positive, classify the same > 90% of the time.
3.6	Comparative reference method	Imaging	Imaging
3.7	Shelf life, stability	36 months at temperatures between 2–30°C; stable for 2 weeks at 50°C.	24 months at temperatures between 2–30°C; stable for 2 weeks at 40°C.
4	Patient access / commercialization		
4.1	Relevant range of cost (price to end-user)	≤ 2 USD	≤ 3 USD per test
4.2	Test pack size	10 or less tests/pack	25 or less tests/pack
4.3	Supply: channels to market	To be determined	To be determined
4.4	Supply: service and support	To be determined	To be determined
4.5	Product registration path	As per local regulations. ISO 13485 for the manufacturing company.	As per local regulations. ISO 13485 for the manufacturing company.

^a^ Tier definitions used as described in Pai et al, 2012 [15]

**Table 4:** TPP for the diagnosis of porcine cysticercosis (various platforms).

	Characteristic or attribute	Optimal	Minimal
1	Context and product overview		
1.1	Indication	Detection of porcine cysticercosis specifically due to *T*. *solium*. To test positive only in the presence of viable cysts. To test negative 4 weeks following effective treatment of cysts. Not to test positive following vaccination.	Detection of porcine cysticercosis specifically due to *T*. *solium*. To test positive only in the presence of viably cysts. To test negative 10 weeks following effective treatment of muscle cysts. Not to test positive following vaccination.
1.2	Intended use (use case; in order of priority)	1- Monitoring *T*. *solium* control interventions. 2- Surveillance and epidemiological studies of porcine cysticercosis.	1- Monitoring *T*. *solium* control interventions. 2- Surveillance and epidemiological studies of porcine cysticercosis.
1.3	Target populations	Any pig population in which *T solium* infection is suspected	Any pig population in which *T solium* infection is suspected
1.4	Target setting for deployment	Cysticercosis endemic or suspected endemic countries	Cysticercosis endemic or suspected endemic countries
1.5	Location of use (infrastructure level)	Basic infrastructure including local animal health care facilities and sub-national laboratories	Diagnostic facilities including research laboratories
1.6	Target user	Veterinarians, veterinary paraprofessionals, and laboratory technicians	Skilled laboratory technicians
2	Design and operational characteristics		
2.1	Format (product presentation)	Technologies providing high specificity and good sensitivity, and that are easy to use. Any traditional or new technology fit for purpose.	Any format meeting the performance requirements
2.2	Portability	Portable	Non-portable
2.3	Target analyte (diagnostic marker)	To be determined. Specific antigen/epitope for the presence of viable *T*. *solium*.	To be determined. Specific antigen/epitope for the presence of viable *T*. *solium*.
2.4	Sample type and quantity	Blood (spots, ear pricks & swabs), oral fluids, ≤ 50 μl.	Serum or plasma ≤ 150 μl
2.5	Sample handling and preparation	One step or none. Samples stable when refrigerated (2–8°C) for 10 days or at -20°C for 12 months.	≤ 7 steps. Samples stable when refrigerated (2–8°C) for 7 days, or at -20°C for 2 months.
2.6	Steps to test result	Few simple steps (< 5 steps)	Many steps (5–10 steps)
2.7	Nature of result	Qualitative	Qualitative
2.8	Time to results	Same day result, < 1 hour.	< 24 hours
2.9	Results records	Test methodology enables clear association between sample ID and result	Test methodology enables clear association between sample ID and result
2.10	Ease of results interpretation	No calculations required	Some basic calculations might be required
2.11	Duration of valid result	≥ 30 min (time from when result is valid to when result is no longer readable/valid).	≥ 10 min (time from when result is valid to when result is no longer readable/valid).
2.12	Throughput	> 300 samples per day	> 90 samples per day
2.13	Equipment/Instrumentation format and complexity	Field-deployable, low complexity equipment.	Basic laboratory equipment such as ELISA readers, centrifuges, incubators, microtiter plate shaker and others might be needed.
2.14	Ease of use	Two or fewer timed steps; instructions should be intuitive, and include diagram of method and results interpretation. Max 2 pages. Instructions for use in English, French, Spanish, Portuguese.	Five or fewer timed steps; instructions should include diagram of method and results interpretation. Max 4 pages.
2.15	Test-specific training requirements	Less than 2 days	Several days
2.16	Supplies needed	Minimal supplies to prepare the sample, packaged as a kit.	Distilled water, pipettes and tips, timer, laboratory material.
2.17	Refrigeration required (storage and transportation)	None required	Reagents to be kept refrigerated (2–8°C).
2.18	Power requirements	Batteries or no power requirements	Some equipment (if needed) such as ELISA readers and others might require mains power, as per manufacturer recommendations (ensure correct voltage and amperage)
2.19	Water requirements	Self-contained kit. Not required.	Distilled or double de-ionized water.
2.20	Operating temperature	10–40°C	20–25°C
2.21	Waste management (hazardous materials/chemicals)	Does not include material that cannot be disposed of in the normal health centres.	Some moderate hazards permitted (e.g. stopping solutions might contain hazardous substances).
2.22	Quality Control	Negative and positive controls included in the kit.	Negative and positive controls included in the kit.
2.23	Calibration	Minimal	Regularly, as per manufactures instructions
3	Performance		
3.1	Species differentiations	*T*. *solium* only	*T*. *solium* only
3.2	Analytic sensitivity / limit of detection	One viable cyst	One viable cyst
3.3	Clinical sensitivity	<50 cysts 70%, > 50 cysts 90%	For ≤50 cysts 50%, for >50 cysts 80%
3.4	Clinical specificity	≥ 98%. Validation should be undertaken in cysticercosis endemic settings. There should not be cross reactions with exposure to, or infection with, other parasite species or show transient positive responses in the absence of mature, viable cysts.	≥ 95%. Validation should be undertaken in cysticercosis endemic settings. There should not be cross reactions with exposure to, or infection with, other parasite species or show transient positive responses in the absence of mature, viable cysts.
3.5	Reproducibility & robustness	Replicate of weak positive, classify the same > 95% of the time.	Replicate of weak positive, classify the same > 90% of the time.
3.6	Comparative reference method	Full carcass muscle and brain dissection	Full carcass muscle and brain dissection
3.7	Shelf life, stability	36 months at temperatures between 2–40°C	24 months at temperatures between 2–8°C
4	Access / commercialization		
4.1	Relevant range of cost (price to end-user)	≤ 0.5 USD per test	≤ 2 USD per test
4.2	Test pack size	100 or less tests/pack	100 or less tests/pack
4.3	Supply: channels to market	Government or aid agencies. Other channels to be determined.	Government or aid agencies, at least one supplier per region. Other channels to be determined.
4.4	Supply: service and support	To be determined	To be determined
4.5	Product registration path	OIE certified & as per local legislation	As per local legislation

Many constructive comments and suggestions were received and incorporated into the final versions. An alternate approach could have involved holding a consensus meeting of experts, however due to limited resources this was not deemed to be a viable option. The consultation via e-mail provided the opportunity for feedback to be extended to an extensive group of contributors who otherwise might not have been able to participate.

The response rate was fifty-three per cent. This was considered to be more than satisfactory as the number and type of invitees was extensive for the size of the taeniasis/cysticercosis field globally. Constructive contributions came from a variety of individuals/organizations and included respondents from Africa, Asia-Pacific and the Americas (Fig 1). The six void responses were 1 from Africa, 3 from Americas, 1 from Asia-Pacific and 1 from an International Organization.

### Taeniasis TPPs (Tables 1 and 2)

Currently available methods for the diagnosis of *T*. *solium* taeniasis lack either sensitivity, specificity, or both (10). Current methods include faecal microscopy to identify parasite eggs, detection of coproantigens or copro-DNA, and detection of specific antibodies in serum. Faecal microscopy fails to identify pre-patent infections and is not species specific, antibody tests continue to test positive after treatment and currently available nucleic acid amplification tests (NAAT) are not suitable for application at point-of-care. Specificity of some tests might be high, such as the coproantigen test described by Guezala et al [16], however the test is not commercially available.

Two TPPs were developed for the diagnosis of *T*. *solium* taeniasis. The rationale for having two tests was that they differ fundamentally in their intended use (surveillance versus monitoring a control program, and monitoring the initial stages in a disease control program versus monitoring later stages) and this connotes important differences in some attributes, such as the use case (attribute 1.2), location of use (attribute 1.5), as well as test specificity (attributes 3.1 and 3.4). A point-of-care antigen (Ag) test (TPP1) was recommended for use at the initial stages of an intervention when specificity for *T*. *solium* would not be critical. The second test, a more specific test (TPP2), was formulated for use in the later stages of an intervention in order to monitor the effectiveness of intervention procedures. If necessary, this second test could be performed in a specialist laboratory (does not need to be a point-of-care test). At the later stages of a control intervention the prevalence of taeniasis would be expected to be very low (prevalence in many highly endemic areas is commonly only 1–2% [17, 18] although higher prevalences have been reported [19, 20], and species specificity would be important to avoid confounded data being obtained due to the presence of other taeniid cestode infections such as *Taenia saginata*. While some respondents suggested that a single test for taeniasis would suffice, it was considered that the different operational requirements for diagnosis of taeniasis in respect of a screening program, as distinct from use in monitoring the effectiveness of an intervention program, would be better reflected in two different diagnostic tests. However, if a point-of-care test with a high level of specificity and sensitivity were developed, it would be suitable for use in both circumstances. The Minimal test attributes for TPP2 were based on a currently existing, non-commercial coproAg test [21], but with some attributes modified to fit the use case.

The draft circulated TPPs were expecting all the tests to be species specific for *T*. *solium*. However, a large majority of the feedback received recommended that the Minimal requirements for a point-of-care test should not include species specificity because it would also be beneficial for the diagnosis and treatment of infections with other *Taenia* spp. Based on this feedback, the final version presented here for the Minimal attributes of a point-of-care test allows detection of other *Taenia* spp. This being the case, the Minimal attributes for the point-of-care test are interrelated, for example species differentiation (attribute 3.1) is linked to the use case (attribute 1.2) and specificity (attribute 3.4) amongst others.

The draft circulated for the Optimal attributes for a point-of-care test (TPP1) was based on a lateral flow assay. In response to the feedback received, the format (attribute 2.1) was changed to a Rapid Diagnostic Test, multiple formats allowed, in order to provide greater flexibility in relation to the technologies that could be adopted.

A number of comments to the draft TPPs concerned the time period after a patient was successfully treated for taeniasis before the test should be expected to revert to being negative. A 6-day period was chosen as being realistic based on the performance of the current coproAg test [21].

Several respondents questioned the age at which humans can be infected with taeniasis. A consensus was reached that, although young children have been occasionally found with *T*. *solium* taeniasis, it was considered unusual to have children younger than 2 years of age infected. Ethical implications of testing young children, including the feasibility of treatment, need to be considered when using the test in the field.

Both the sensitivity and specificity for the taeniasis test TPPs (attributes 3.3 and 3.4) circulated as drafts were altered in the final versions based particularly on feedback that highlighted a need for very high test specificity for monitoring control programs. Specificity was prioritized for all taeniasis tests, except for the Minimal attributes point-of-care test, in which sensitivity was prioritized considering that the test would be used for surveillance, and not for monitoring an intervention.

**Fig 2:**
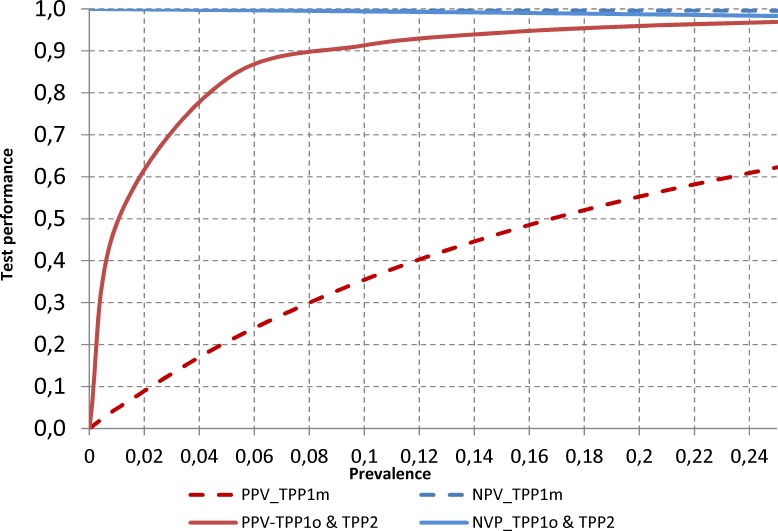
Positive predictive values and negative predictive values of TPP1 and TPP2 tests for human taeniasis. Graph showing data within a prevalence range 0–25%. 0 = optimal; m = minimal. For TPP attributes see Tables 1 and 2.

While sensitivity and specificity are inherent test characteristics, they should be defined in the TPP taking into account the expected prevalence of the condition to be diagnosed in the conditions of use, which will determine the predictive value of a positive (PPV) and a negative (NPV) test. There is limited data about the prevalence of *T*. *solium* as much of the published information refers to taeniasis without defining the species. However, the starting prevalence of *T*. *solium* taeniasis is expected to be around 1–2%, and up to approx. 5% [17–19] although some papers mention hotspots of up to 26% [20]. Within the 1–5% prevalence range, the PPV of the optimal TPP1 and both the TPP2 tests will be 49%-83% (specificity 99%) and the NPV 100% (sensitivity 95%). The minimal TPP1 test too will have 100% NPV (sensitivity 99%) but its PPV will be much lower 5%-21% (specificity 80%) (Fig 2). This means that a test with sensitivity of 95% or more is adequate within the expected prevalence range, and that, if it is also 99% specific, it would be suited for both the initial and the later stages of an intervention.

The reference test (attribute 3.6) selected for *T*. *solium* taeniasis [14] was chosen on the basis that the test has high sensitivity and specificity, has shown to detect immature tapeworms and has been validated extensively.

The relevant range of cost (price to end-user, attribute 4.1), was based on what was considered realistic based on the feedback provided, but no real cost analysis was done.

### Neurocysticercosis TPP (Table 3)

Current tests for the diagnosis of NCC include antibody detection (enzyme-linked immunoelectrotransfer blot, ELISA) and antigen detection (ELISA) tests. A challenge with the currently available tests is that a proportion of people living in endemic areas might test transiently positive on either antibody or antigen detection tests, apparently in the absence of mature or clinically-relevant cysticercosis [22–25]. Antibody tests do not differentiate between patients having viable cysts from those with non-viable cysts.

Comments were received and discussions held on the need to include also detection of antibodies, in order to detect patients with only non-viable/calcified cysts. Viable cysts degenerate over time, and some may eventually be calcified [17, 26]. In cases where cysts become non-viable, inflammation, degeneration and possible calcification occur, although precise staging is not always clear because changes in and around the parasite occur as a continuum [8] and this is reflected in the performance of currently available diagnostic tests (Table 5). Overall, consensus was reached that the priority was for a point-of-care test to be used in symptomatic patients in order to detect those with viable cysts who needed to be referred for imaging and further management, including treatment with anthelmintics. Anthelmintic treatment for NCC should not be initiated on the basis of a serology result alone, as cyst development/inflammation and the presence of oedema need to be determined prior to the initiation of treatment [6]. It was considered that differential diagnosis of symptomatic patients with non-viable cysts was less of a priority because their treatment would not be significantly different to other cases of epilepsy. Similarly, detection of non-symptomatic cases of subjects with viable cysts was not considered a priority as many lesions may remain asymptomatic (between 50–80% of those affected) or resolve spontaneously without symptoms [6, 27]. As many NCC patients are poor and live in remote rural areas, priority was given to the detection of only symptomatic patients who could be helped by anthelmintic treatment of viable cysts and for whom their investment in attending a medical center for brain imaging and treatment warranted the expense. Test attributes were selected to avoid identification of other NCC patients for whom the expense of travelling to a medical center and medical imaging would not greatly assist their medical prognosis.

**Table 5:** Characteristics of *Taenia solium* cysticerci in the brain with respect to cyst viability and diagnostic parameters.

Cyst stage^a^	Viable, no inflammation	Early inflammation	Late inflammation	Cysticercal granuloma	Calcified
Parameter					
Cyst viability	Yes	Yes	?	No	No
Circulating antigen	++	++	+/-	-	-
Circulating antibody	++	++	++	++	+/-
Brain inflammation	-	+	++	++	- ^b^
Symptoms	Some or potential	Yes	Yes	Yes	Some

^a^ Cyst stages are not discrete; they occur as a continuum; the stages described are relatively arbitrary and for discussion purposes.

^b^ Recent studies shown that when calcified cysts are implicated in seizures, about 30–50% are associated with surrounding edema likely due to an inflammatory process [26].

The analytical sensitivity/limit of detection (the smallest amount of substance in a sample that can accurately be measured by an assay) was a frequent topic of discussion. A consensus emerged that the Optimal attribute should specify to detect patients with a single intracranial cysticercus, including both intraparenchymal and extra-parenchymal cysts. However, several opinions were received regarding the Minimal requirements. Eventually a compromise was reached to prioritise the detection of high-risk patients, defined as patients with 5 or more parenchymal cysticerci, or with a single ventricular or subarachnoid cysticercus.

Respondents considered that it would be useful if a test could be used to monitor the effectiveness of anthelmintic treatment of NCC, particularly in specific scenarios such as patients with subarachnoid NCC [28]. Hence this was added to the Optimal use case. This necessitated the test being quantitative or at least semi-quantitative, and appropriate changes were made in the final TPP.

An important and complex issue was how to deal with the existence of cases where transient serologically positive responses occur (both for antibody and antigen) in people living in *T*. *solium* endemic areas. These responses could potentially be due to people being exposed to *T*. *solium* eggs, with the parasite possibly undergoing early development sufficient to induce a detectable serologic response, but not completing development and hence not causing detectable or symptomatic NCC. However, the precise cause of these transient responses has not been determined and it could be due to some cause unrelated to *T*. *solium*. This is particularly the case for the currently used test for circulating antigen which is known not to be specific for *T*. *solium*. In the case of the Minimal attributes for NCC, the existence of transiently positive reactions is included and, based upon the feedback that was received, a consensus was reached that a positive test would require to be repeated with a second sample collected after three months in order to confirm a case of persistent NCC. However, depending on the history and severity of the signs, the physician might decide to refer the patient immediately without repeat testing.

Optimal sensitivity and specificity was reviewed, increasing the sensitivity and decreasing the specificity suggested in the draft, based on the need to detect the patients to be referred, but also accounting that the test would be used in a pre-selected population (symptomatic patients), so the test would still have a high positive predictive value.

### Porcine cysticercosis TPP (Table 4)

The definitive method for the diagnosis of porcine cysticercosis is detection of cysts with a full carcass muscle and brain dissection; this is both time consuming and expensive, particularly in the case of light infections. Serological tests (both for circulating antigen and antibody) are available but specificity can be problematic when used for monitoring a control program. It is important that tests do not cross react with other taeniid cestode parasites to which pigs are likely to be exposed. Additionally, many pigs in endemic areas that are serologically positive have no cysts at necropsy [29–31]. It is therefore critical that any test developed is validated in endemic areas, in pig populations similar to the ones that would be targeted in a control program.

The Minimal attributes were based on a currently available commercial antigen detection test for *T*. *solium*, with some attributes modified to fit the use case. The occurrence of transient positive responses in animals that do not have mature cysts were not considered acceptable, unless it could be demonstrated unequivocally that these responses only occurred in animals exposed to *T*. *solium* and are not induced by any other cause such as exposure to any other parasites.

Discussions were held with a number of experts on the period over which the test would revert to being negative after a successful chemotherapy. The drug most commonly used to treat porcine cysticercosis is oxfendazole [32, 33]. There is limited published evidence on the dynamics of circulating cysticercal antigen after oxfendazole treatment [34, 35]. Sikasunge et al. [34] found a statistically significant reduction in circulating antigen level at 8 weeks post-treatment. For this reason, a Minimal period of 10 weeks, and an Optimal period of 2 weeks for the test to revert to negative were considered appropriate.

Oxfendazole is highly effective for the treatment of cysticerci in muscle tissues, although efficacy is limited in the treatment of brain cysts [36]. It is unclear if cysticercal antigens in pigs cross the brain-blood barrier. Dorny et al [37] found that one pig having 16 viable cysts only in the brain was negative for the presence of *T*. *solium* circulating antigens, however there are few data on serological responses in animals having only brain cysts. With these considerations in mind, in order to provide flexibility in the development of diagnostics for porcine cysticercosis the TPP’s Minimal indication for treated animals (attribute 1.1) was worded in a way such that it would not be restricted if the test were positive in animals having only viable brain cysts.

A consensus was reached based on feedback from respondents that a qualitative test would be fit for purpose, hence this was changed from the proposal for a quantitative test that was included in the draft TPP which had been circulated. Analytical sensitivity was defined at 1 cyst for the Optimal attribute. A variety of views were expressed in this respect for the Minimal attribute, and a decision was taken to define the limit of detection for this test to also be 1 cyst.

Sensitivity and specificity were also reviewed based on the feedback provided. Based on the use case, priority was determined to be specificity and hence this was maintained at a high level as per the original draft. Sensitivity was reviewed down, because of the challenges in achieving good sensitivity in an antigen test. A test with a relatively low sensitivity would nevertheless be useful in monitoring progress of control efforts, so long as the specificity was high.

## Conclusion

The TPPs were finalized based on the contributions of many subject matter experts serving different roles in academia, government, industry, NGOs and international organizations. Often a variety of views were expressed about a particular test attribute and, through additional discussions, in most cases a consensus was determined and this then formed the attribute included in the final TPPs. Each TPP is likely to need to be reviewed and updated in the future, as knowledge and technology progresses. It is expected that the TPPs presented here will assist researchers and diagnostic developers in guiding their efforts and ultimately development of the tools needed to assist in the control of *T*. *solium* transmission and a consequent reduction in the incidence of human NCC.
